# 5G and IoT Based Reporting and Accident Detection (RAD) System to Deliver First Aid Box Using Unmanned Aerial Vehicle

**DOI:** 10.3390/s21206905

**Published:** 2021-10-18

**Authors:** Monagi H. Alkinani, Abdulwahab Ali Almazroi, NZ Jhanjhi, Navid Ali Khan

**Affiliations:** 1 Department of Computer Science and Artificial Intelligence, College of Computer Sciences and Engineering, University of Jeddah, Jeddah 21959, Saudi Arabia; malkinani@uj.edu.sa; 2 Department of Information Technology, College of Computing and Information Technology at Khulais, University of Jeddah, Jeddah 21959, Saudi Arabia; aalmazroi@uj.edu.sa; 3 School of Computer Science and Engineering, Taylor’s University, Subang Jaya 47500, Malaysia; navidalikhan@sd.taylors.edu.my

**Keywords:** 5G, IoT, edge computing, unmanned aerial vehicles, intelligent transportation

## Abstract

Internet of Things (IoT) and 5G are enabling intelligent transportation systems (ITSs). ITSs promise to improve road safety in smart cities. Therefore, ITSs are gaining earnest devotion in the industry as well as in academics. Due to the rapid increase in population, vehicle numbers are increasing, resulting in a large number of road accidents. The majority of the time, casualties are not appropriately discovered and reported to hospitals and relatives. This lack of rapid care and first aid might result in life loss in a matter of minutes. To address all of these challenges, an intelligent system is necessary. Although several information communication technologies (ICT)-based solutions for accident detection and rescue operations have been proposed, these solutions are not compatible with all vehicles and are also costly. Therefore, we proposed a reporting and accident detection system (RAD) for a smart city that is compatible with any vehicle and less expensive. Our strategy aims to improve the transportation system at a low cost. In this context, we developed an android application that collects data related to sound, gravitational force, pressure, speed, and location of the accident from the smartphone. The value of speed helps to improve the accident detection accuracy. The collected information is further processed for accident identification. Additionally, a navigation system is designed to inform the relatives, police station, and the nearest hospital. The hospital dispatches UAV (i.e., drone with first aid box) and ambulance to the accident spot. The actual dataset from the Road Safety Open Repository is used for results generation through simulation. The proposed scheme shows promising results in terms of accuracy and response time as compared to existing techniques.

## 1. Introduction

Road traffic accidents (RTAs) are becoming more common nowadays, as evidenced by the fact that the number of accidents is increasing on a daily basis. Increasing populations increase the number of vehicles on the road, increasing the likelihood of accidents occurring on the road. Every year, according to the World Health Organization (WHO), traffic accidents result in 50 million injuries and 1.35 million fatalities [1]. Accidents involving automobiles are now the eighth most common cause of death, up from ninth in 2015. If safety measures are not undertaken, this rating could jump from eighth to fifth in the near future, according to the Association for Safe International Road Travel (ASIRT). Every country spends between 1 to 2 percent of its yearly budget on road accidents, according to ASIRT [2].

In recent years, even in developed countries with flawless road safety regulations, mortality in traffic accidents have been on the rise [3]. The lack of immediate help to save a life is one of the most prominent causes of death in a road traffic accident. Some various methods and techniques can assist in minimizing the frequency of traffic road accidents and saving lives. Using new technologies with diverse strategies in our daily lives may be solved in this modern world evolving with new technologies day by day. The vision of 5G and the Internet of Things (IoT) can enable unforeseen applications, such as smart healthcare, smart cities, intelligent transportation, and many others in today’s world [4]. Various technologies support these applications and meet needs like high data rate and low latency, among others [5]. These applications use a variety of sensors to collect data from the environment every minute and exchange it with one another.

Based on this information, further action is taken. In today’s environment, the need for accident detection and reporting to relevant authorities and family is vital. Accident detection and tracking, as well as timely notification of an accident to the emergency department. This will result in lives being saved and injured persons being rescued.

As a result, this study presents a low-cost 5G and IoT-based reporting and accident detection system. The proposed method is categorized into two phases: identification of accidents and reporting of those accidents Multiple smartphone sensors are employed to identify an accident, including GPS, accelerometer, microphone, and pressure sensors. An android app is developed to gather data from the sensors and send it to the edge cloud for processing. Threshold analysis is used to determine the cause of an accident. When an accident is detected, the nearest hospital, police station, and family are notified.

### 1.1. Our Contribution

- The development of an Android application that collects data from sensors;
- Development of website which is connected to edge server to identify the accident through threshold analysis;
- Calculating and defining threshold values for accident identification;
- Reporting and notification to nearest hospital, police station and concerned person.

### 1.2. Paper Organization

The remaining paper is arranged as follows. Related work is presented in Section 2. Section 3 presents the proposed architecture. Section 4 presents accident detection methodology. Section 5 presents simulation result and comparison. Finally, Section 6 presents the conclusion and future work.

## 2. Related Work

The rising incidence of car accidents is posing a serious problem for our community, and therefore immediate action is required. The Internet of Things (IoT) is one of the most promising technologies in the field of intelligent transportation. Many of the researchers used IoT to help with smart transportation. Furthermore, because of their vast range of movement in any direction, UAVs play an important role in these applications [6]. UAVs can fly at lower altitudes in any direction, allowing them to navigate areas that are not normally accessible by humans [7]. Due to these characteristics of UAVs, delivering a first aid kit to an accident site quickly is quite beneficial [8].

In paper [9], a framework is presented by the researchers having two phases. In the first phase, the system detects an accident and notification system while it provides a management system for an ambulance in the second phase. The routing scheme informs the ambulance about the efficient route. The presented technique is suitable for the road junctions having traffic signals. However, the method does not apply to traffic signals. In paper [10], the researchers presented an accident management system to find out the trade-off while considering different measures such as false delivery, high cost and non-probability. The proposed scheme is not suitable to limit due resources. The technique used a severity scale to measure the impact of an accident.

In paper [11], the authors presented a system to detect an accident at high and low speed by considering different scenarios. In the high-speed scenario, if the value of acceleration becomes more significant than 4G, it is declared an accident with the help of a smartphone application. However, in few cases, it activating of false alarm notifications meanwhile mobiles are subjects. In paper [12], the researchers proposed a notification system based on a call by utilizing different components, such as Xbee shield, GPS, Seeduino, and Wi-Fi module. The crash sensors are used to detect an accident which leads to less accurate results.

In paper [13], The researchers provided a strategy for detecting accidents that are primarily caused by those who had consumed alcohol while driving. According to the author, different sensors, including alcohol sensors, heartbeat sensors, and touch sensor interfaces with a Raspberry Pi, were used in this system. The approach that has been described is only appropriate in the scenario of drunk driving.

In paper [14], the researchers study the behavior of the driver by using IR sensors to analyze the eye blinking. With the help of an accelerometer, the driver’s head motion is monitored. The accelerometer is fixed on the driver’s forehead to calculate the angles made by the movement of the head. The technique would not be comfortable by attaching the accelerometer every time with the forehead. Additionally, only the driver’s behavior to detect an accident may be inaccurate.

In paper [15], the authors discussed a technique for accident detection by using hardware with different sensors. In this technique, the severity of an accident is identified with the help of generated results. In paper [16], the researchers detect an accident with the use of a fitted unit in the car. This unit is fitted as push-on switches that observe any triggers and obstacles. The unit microcontroller (AT8952) helps turn on the beeper by following any trigger or barriers. However, the technique may not work if the driver forgets to turn on a switch.

In paper [17], the researchers presented a system that detects an accident by observing the condition of a car engine and informing the user about smoke or any flame seen in the car engine. The system can effectively monitor all the abnormal conditions that occur in the car; however, the presented system does not consider accident detection strongly.

In paper [18], the researcher presented an accident detection system and notifies the concerned number in case of an accident. In this technique, the system report to the concerned number instead of emergency service. However, due to limited resources and implementation, the system is unpolished.

In paper [19], the researchers presented a technique which has the ability of auto-detection of collision with the help of accelerometer and forwarded the information to emergency service with the help of a global system for mobile (GSM) messaging. However, the system is based on one sensor, which may not generate accurate results. In paper [20], the authors presented a method of vehicle accident detection and tracking with the help of GSM and GPS. The push turns on switches detect an accident and track the location with the help of GPS to inform the user-define number by using GSM service.

In [21], EARVE, an architecture framework for vehicle-to-edge applications, is presented. The system takes use of the low latency of edge servers to enable real-time emergency detection and alerting. EARVE’s layered design provides servers at the street, neighbourhood, and city levels, allowing for a range of uses at various time and spatial scales. The findings reveal that EARVE reduces the latency of AR applications in car networks when compared to cloud solutions. In [22], an innovative vehicle-to-multi-edges (V2Es) communication paradigm is given to increase vehicular networks’ computational and communication capacity. A multi-agent RL algorithm is deployed to learn the real-time communication status between vehicles and a large number of edge nodes and make suitable job offloading and edge caching decisions. Simulations have shown that the suggested method may effectively minimize average service delay and energy usage. In [23], to conduct research into different vehicular computing architectures, as well as prospective data analytics technologies and their interaction with vehicular computing systems, a thorough examination of distributed vehicle computing systems is provided, including centralised cloud computing, vehicular cloud computing, and vehicular edge computing.

Table 1 shows the summary of different techniques with prominent features and limitations. In addition, the implemented methods and tools are also discussed.

## 3. Proposed Architecture

**Figure 1:**
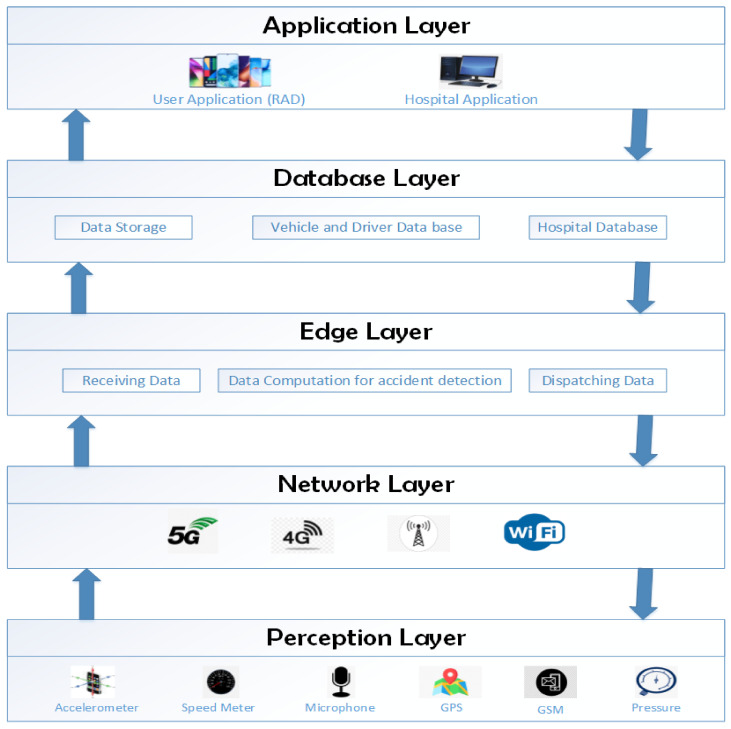
Reporting and Accident Detection (RAD) architecture.

To keep in mind the above limitation in the detection of accidents systems. We are presenting a novel reporting and accident detection system (RAD). Our system is based on an Android smartphone that does not need any special hardware to decrease the cost. The data processing is based on cloud computing. The RAD architecture is based on layers which are shown in the Figure 1.

The RAD architecture is composed of five distinct layers: apps, database, cloud, network, and perception. The perception layer in the described RAD architecture is responsible for communicating with the smartphone’s sensors. The perception layer’s principal function is to gather data from the sensors. The collected information is based on sound, speed, pressure, gravity, and vehicle location.After data collection, it is passed to the network layer for processing. The network layer’s function is to connect the edge to the perception layer. The network layer is based on 4G, 5G, or Wi-Fi to transfer the perception layer to the cloud layer. The presented algorithm is embedded in the edge layer, detecting the accident and performing the analysis based on a defined threshold. If any accident is detected, the nearest hospital and ambulance are informed, and data are forwarded to the database layer. Finally, the database layer saves the data in the database (i.e., accident information, driver information, vehicle information, and hospital information). Collected data are then forwarded to the application layer for further process with the help of smartphones and web-based systems for the driver and hospital.

The driver installs the application on their smartphone from the Google Play Store. After installation, the applicant registers the required information needed and then the users are free to use the application. With every journey, the applicant turns on the tracking process. First, the sensors embedded with the smartphone start data collection and send it to the cloud. Then, the edge starts accident detection by using the provided information.

In the proposed scheme, every car connected to a smartphone is assumed. Every smartphone is based on four sensors; noise sensor (i.e., microphone), pressure sensor, speed sensor, and accelerometer. The android phone with the sensors mentioned above is continuously used for data collection to perform experiment evaluation. In order to detect a mishap, the smartphone transmits the information to the cloud, where it is processed by the edge, which determines whether or not an accident has occurred. This value is predetermined; if the sensor data collected is more than this value, then an accident has occurred. When the predefined conditions are qualified, an alarm is activated, and the alert notification is given to the driver. To avoid false reporting, if the driver cancels the alarm before it goes off, the hospital will not be reported. If the driver has taken no action within 10 s, the nearest hospital and UAV ambulance are informed with edge service help. The edge service informs the nearest ambulance about the location of the accident for the rescue operation. The cloud service maintains the database of hospitals, cars and ambulances. The proposed scheme is based on two phases. (I) Detection of accident; (II) Notification phase.

The primary goal of the model is to provide an architecture that allows and delivers the goals listed below.

- To enable vehicle to infrastructure (V2I) communication directly.
- To develop a system with cost-effective;
- To improve the accident detection accuracy;
- To decrease the false reporting information.

**Figure 2:**
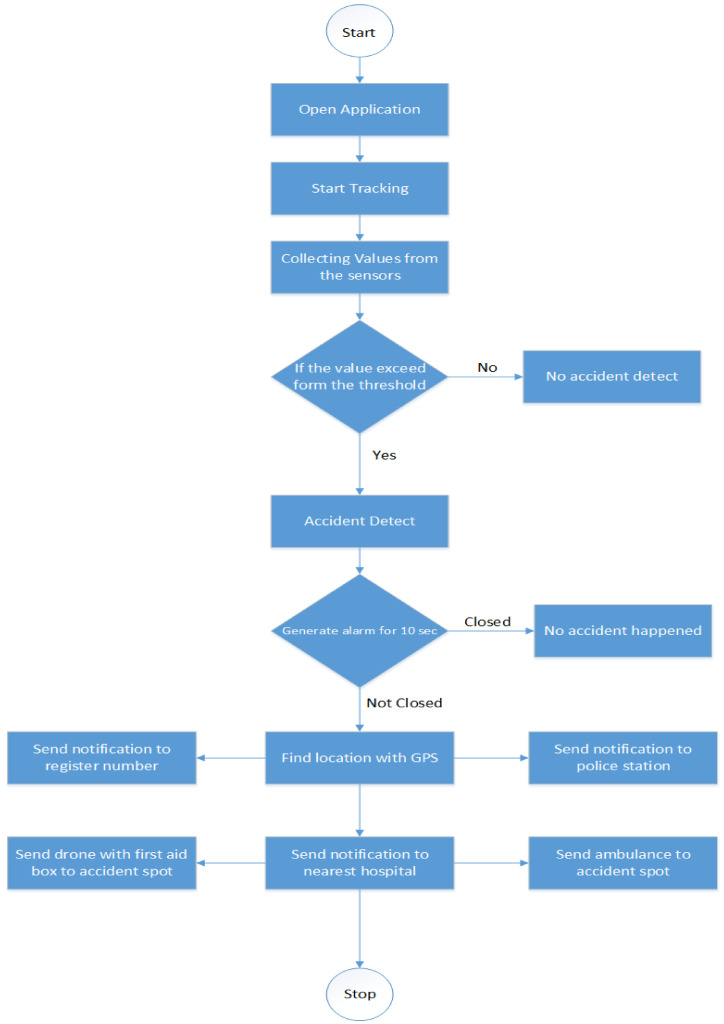
Flowchart of proposed system.

Figure 2 shows the flow chart of the proposed architecture.

### 3.1. Accident Detection Phase

Accident detection aims to avoid unfortunate events during driving that cause injury or damages and minimize the death ratio during traffic accidents. In this phase, different components, such as microphone, pressure sensor, accelerometer, and GPS are used to identify the accident occurrence. Further details of these components are as follow.

#### 3.1.1. GPS Technology

GPS technology aims to extract the positional data (i.e., global navigation satellite system). GPS helps to find out the vehicle position and transmit that data via the system. The provided data are useful to calculate the vehicle speed. The speed of a car helps to identify the accident accurately.

#### 3.1.2. GSM Technology

This technology is used to transmit mobile data (i.e., message to police station, hospital, and concerned person). The purpose of the message is to deliver a notification about the accident for additional confirmation.

#### 3.1.3. Microphone

This component is useful to sense the sound. The flag of an accident is raised when the value of sound beats from the defined threshold value (i.e., 140 dB). Subsequently, we use a pressure sensor and accelerometer to identify an accident better, leading to better accuracy. A smartphone with a microphone leads to better accuracy by decreasing the probability of false positives.

#### 3.1.4. Accelerometer

The smartphone accelerometer sensor is helpful to observe the acceleration force. This accelerometer is one of the significant components for accident identification. The accident flag is raised when the acceleration force becomes high than 4G [28]. It should be kept in mind that G-force data are not enough to detect an accident accurately. Additionally, the 4G threshold value is derived via experimentation and secondary research.

#### 3.1.5. Pressure Sensor

The pressure sensor collects data about the vehicle’s pressure during the collision and before collision continuously. The accident flag is raised when the pressure value exceeds a predefined threshold value (i.e., 350 Pa). Thus, the information of pressure helps us identify the accident occurrence and reduce false reporting of an accident.

### 3.2. Notification Phase

Notification in time about accident occurrence is a crucial phase in this phase message immediately forwarded to hospital and emergency rescue teams for further action. The system gets the location with the help of GPS in a smartphone after an accident takes place. The information related to the accident, such as the value of speed, pressure, noise and location, is forwarded to edge with the help of 4G/5G. The edge server has a hospitals database and finds out the nearest hospital with a mapping service. The nearest hospital is informed about the accident along with owner information and the location of the accident. The existing database is updated about an accident.

### 3.3. Vehicles Databases

**Figure 3:**
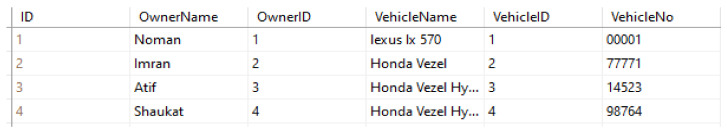
Vehicles database.

This database contains information about the vehicles. For example, owner name, owner ID, vehicles Name, vehicles name, and vehicles ID. Figure 3 shows the vehicles database.

### 3.4. Hospitals Databases

**Figure 4:**
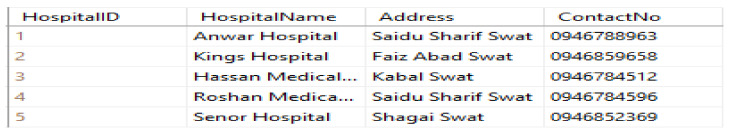
Hospitals database.

For emergency services, the system needs information about hospitals. After accident detection, the system forwarded the notification about an accident to edge cloud, and the edge cloud informs the nearest hospital for further action. Figure 4 shows hospitals database.

## 4. Our Proposed Methodology

In this section, we describe the proposed system model. The proposed system is based on two key mechanisms. An Android application for smartphone and web-based systems. The android application helps us collect data, such as pressure and noise with the help of the accelerometer and smartphone microphone. Based on these values accident is identified.

- Establish a connection

The Android application is installed by the user and activated with internet access (i.e., Wi-Fi/4G/5G). With the help of the Android application, the smartphone collects the data of pressure sensor, accelerometer and microphone, and GPS data.

- Detection of accident

The equation for detection of an accident in the [40] is used. Where DA is flag pointer of accident occurrence. In the equation:

*AC* is a value of acceleration that is noticed with the help of a smartphone;

*Noise* is a value noise noticed with the help of a smartphone by using a microphone;

*SVP* is a variation of speed after a specific period, which is helpful to identify the accidents at low speed;

*Threshold* of Accident (TA) is the value defined (i.e., 1.5) that accident detected;

*Speed (S)* is a value of speed, calculated by G-Force;

*Threshold for low speed* (TLS) is the value defined (i.e., 3) that accident detected at low speed;

*MPT* is a maximum period time to consider an accident at low speed.

The cloud identifies the accident with the help of Equation (1) by processing the collected data. The alarm is activated when the accident is identified. There are 10 s with the user to deactivate the alarm to avoid false accident reports. If the alarm is not deactivated within 10 s, the nearest hospital is informed with an emergency message for further action. Algorithm 1 describe detection of accident.

Notification when an accident is confirmed, the location of an accident is found out with the help of GPS. In the proposed scheme, Google Map is used to find out the location of an accident. The system forwarded the information of vehicle, location and passengers by utilizing 4G/5G/Wi-Fi to the nearest hospital for immediate action.

(1) $$DA=\left\{\begin{array}{cc} 1, & if\left(\frac{Noise}{140}+\frac{AC}{4G}+\frac{Pressure}{350}\right)\geq \left(TA\right)\Lambda \left(speed\right)\geq 24\;\mathrm{km}/h,\\ 1, & if\left(\frac{Noise}{140}+\frac{AC}{4G}+\frac{Pressure}{350}\right)\geq \left(TLS\right),\\ 1, & if\left(\frac{Noise}{140}+\frac{AC}{4G}+\frac{Pressure}{350}\right)\geq \left(TA\right)\Lambda \left(ElapsedTime<MPT\right),\\ 0, & otherwise. \end{array}\right\}$$

**Algorithm 1:** Accident Detection
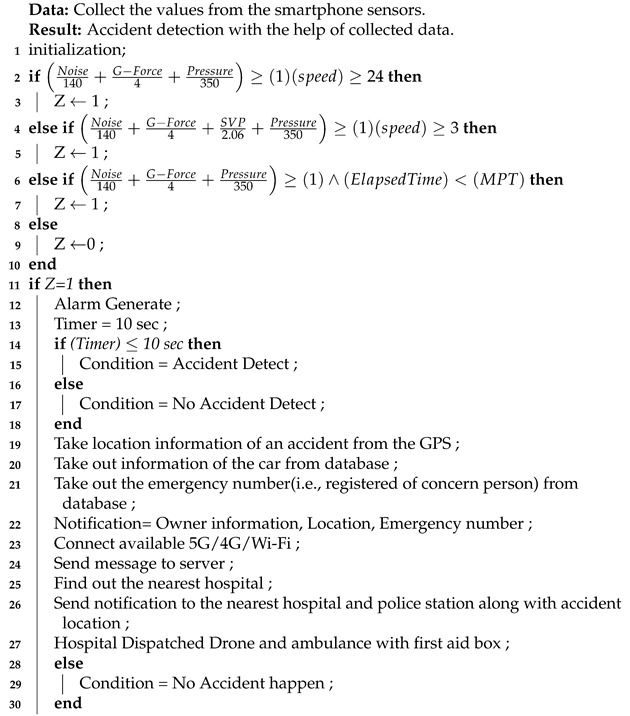


### 4.1. Implementation of System

**Figure 5:**
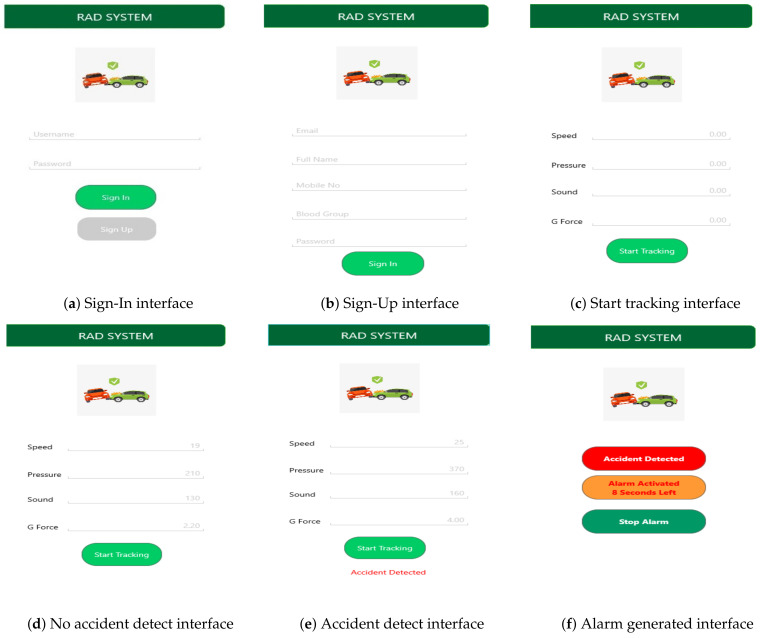
Andriod application interfaces.

As mentioned earlier, the proposed system has two phases: the android application is a smartphone used to detect an accident, while the web-based system is used for hospital notification. Implementation of the accident detection phase Java language is used for programming, and the android studio is used for android application development. After installation of an application on a smartphone, the user needs to register with different information. Once the registration process is completed, the applicant can log in to the system with their user name and password. After logging into the system, the user clicks on the start tracking button to transmit and record data. The application continuously collected the data from the sensors of the smartphone and forwarded it to the edge. If the value exceeds the defined threshold, the edge identifies the accident and generates an alarm for 10 s. Figure 5 shows the interface of the Android application. The android application contains the mentioned below activates.

- Account management;
- Collection of data;
- Activation and deactivation of accident detection;
- Tracking of an accident;
- Alarm cancellation.

### 4.2. Implementation of Notification Phase

After identifying an accident, the nearest hospital is notified about an accident with the help of edge based on a web application. ASP.NET is used to develop a web application. The hospitals use this application to identify emergency conditions. In the event of an accident, the website is notified and receives information regarding the accident. The website shows information about an accident, such as drivers information, vehicle information, and accident location. HTML, CSS, and bootstrap are used to develop a website and Microsoft SQL to store accident data in the database. For location tracking, Google Maps is used to identify the accident location on a map.

## 5. Simulation Results

Due to damage issues, safety and cost, the proposed scheme cannot be implemented in a real scenario. However, we did the simulation for the proposed system in a controlled environment. The proposed system records acceleration based on the value of speed, pressure, and noise at a possible high rate. The collected data are forwarded to edge for accident identification.

### 5.1. Threshold Evaluation

**Figure 6:**
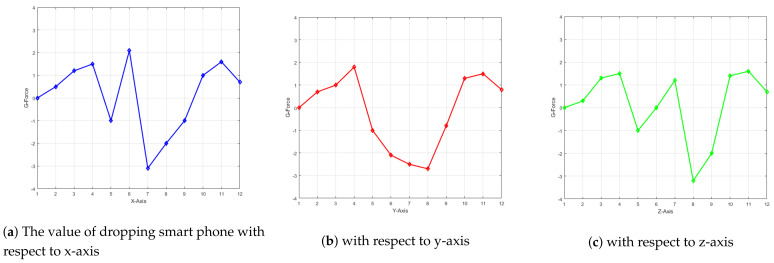
The values of G-Force while dropping a smartphone.

For threshold evaluation, we calculate the G-force values shown in the Figure 6. Where the real drivers and vehicles are used to drive at different speeds. It has been stated that maximum force experience is not more than 3.3 G.

The other factors, such as G-force, speed, and pressure are also calculated to test android application.

### 5.2. Benchmark CADANS

This section presents our simulation results which are the accuracy and response time of our android application. ACC simulator is used to calculate the accuracy and response time. The results are compared with care accident detection and notification system (CADANS) [32], which is based on smartphone sensors (i.e., accelerometer, microphone, and GPS) for accident detection. We considered four sensors proposed by RAD system and evaluated the behavior of CADANS with three sensors. The results show that RAD perform well as compared to CADANS in term of accuracy. In some cases, accidents are not detected by CADANS while the accidents have occurred. In the simulator, we used ADSim, using a mobile ad-hoc network (MANET) and threads to handle many vehicles. Each sensor has a set of classes and own get, which generate values in every second. We did a simulation for three minutes to obtain the results and analyze them. Simulation parameters are given in the Table 2. The accident formula is executed with different values of each sensor to identify the accident.

**Figure 7:**
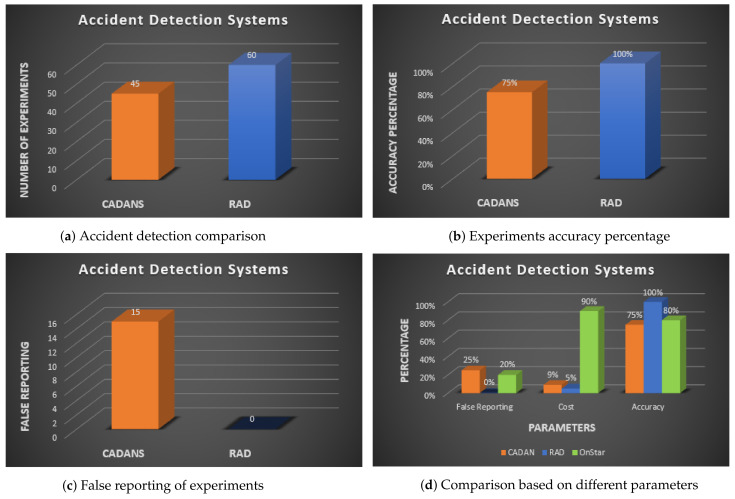

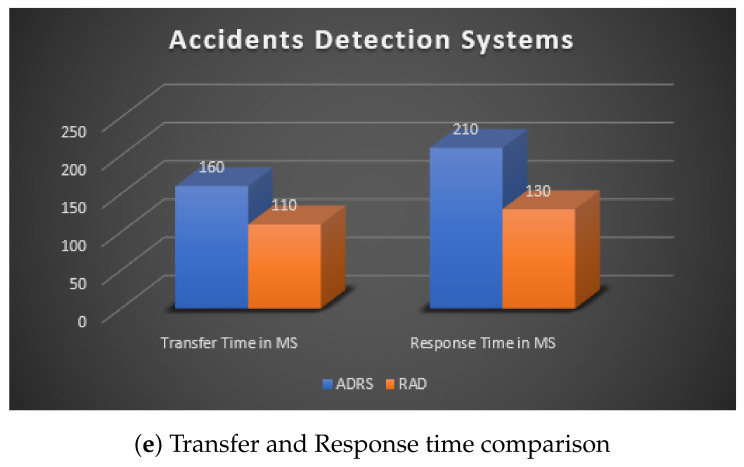
Simulation results.

The simulation was run for three minutes. In this period, 60 accidents happened, and all of them were detected by the proposed system RAD, where only 37 of them were detected by CADANS accurately. CADANS also generated false reports compared to RAD. CADAN detects accidents where the G-value is less than 3 G (i.e., caused due to smartphone dropping). CADANS reports 60 accidents where 14 of them were not actual accidents. The pressure sensor data helped RAD system to detect accidents accurately and decrease the false report probability. Figure 7a–c shows the simulation results.

### 5.3. OnStar System as a Benchmark

The OnStar system [41] is used for the comparison with the proposed system RAD. OnStar system is developed by General Motors (GM) for assistance on roads. The system is based on sensors embedded in the vehicle which detect an accident and inform the rescue team. OnStar system is hardware base which is embedded in-vehicle sensors. The system only work in GM vehicles and cannot be embedded in other cars. Figure 7d shows the simulation results.

### 5.4. Benchmark with ADRS

The ADRS system [40] is selected for comparison in terms of response time. The ADRS uses the same sensors as used in RAD system. ADRS is based on cloud computing which is far as physical, which can lead to high latency. As a car accident is a severe case, therefore real-time detection is very important. RAD system is based on edge computing which processes the data near to the foundation and prioritizes the traffic in the network. Edge computing minimizes data flow from and to the primary network, leading to low latency and high overall speed. Figure 7e shows the simulation results.

### 5.5. Evaluation Using FODR Dataset

For more experiments, we used the FODR dataset for the detection of an accident. The Find Open Data repository is available at https://www.data.gov.uk, accessed on 30 January 2019. The values of speed and noise are obtained from the actual accidents. We experimented with three different scenarios by considering various sensors.

*Scenario 1:* In this scenario, the real extracted value of speed is compared with the system having one sensor [42]. The system is unable to detect an accident at a speed less than 24 km/h.

*Scenario 2:* In this scenario, the system having two sensors is considering [32]. The value of speed and noise is considered. The system is unable to identify the accident at a lower speed. The system may have detected an accident when the speed was higher than 24 km/h while no accident was real. In another case, if the noise value exceeds the pre-defined threshold, it may not detect an accident, while the accident may have happened in fact.

*Scenario 3:* In this scenario, multiple sensors, such as speed, accelerometer, pressure, and noise, are used to detect an accident. Using multiple sensors improves the accident detection accuracy and decreases the false positive and false negative chances. It may also help to detect an accident that is missed by other systems at a lower speed.

**Figure 8:**
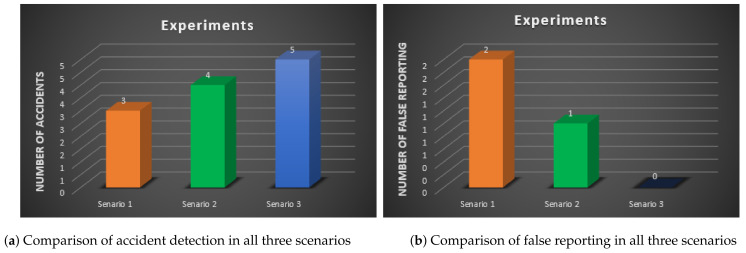
Simulation results.

In Scenario 1, only three accidents are detected out of five. In Scenario 2, only four cases are detected out of five. Finally, in Scenario 3, all five accidents are detected out of five. In Scenario 3, the accuracy of the proposed system is 90%. Figure 8 shows the accuracy and false reporting of all three scenarios.

## 6. Conclusions and Future Work

The number of vehicles on the road in today’s society is steadily increasing. As a result, the number of accidents is rising. Despite the fact that there are numerous technologies for accident detection, the death rate continues to climb. The inaccuracy of accident detection and poor notification methods are to blame for the late response to catastrophic accidents. The lack of accessibility to realistic retrofitting solutions, as well as economic issues exacerbates the problem. We introduced 5G and IoT-based technologies to identify an accident to address the aforementioned challenges. Using several sensors has been found to improve the accuracy of accident detection. The technology recognizes the accident and the nearest hospital in real-time. It immediately sends an emergency notification to the nearby hospital and a family member or friend. Using the smartphone’s sensors, it is possible to detect an accident and alert the user. Our proposed technique has been found to lessen the number of false positive reports of accident detection. For our system to work, it needs to be connected to the internet. We will soon put our system to the test in a real-world scenario. As a result, the privacy and security of the drone network are critical. We intend to tackle the privacy and security issues in the near future because the system requires complete privacy and security.

## Figures and Tables

**Table 1:** Review of existing schemes findings.

Ref	Features	Limitations	Performance Measures	Simulators
[24]	Accident detected through GPS speed	Accident report is not accurate	Time Accuracy	GPS and GSM modem
[19]	Accelerometer is used to detect accident	Failure due to single point	Time Accuracy	Real Implementation
[25]	Rescue system with accident detection	Manual system	Efficiency	Real Scenario implementation
[26]	Detection of accidents at point of intersection	Valid only at point of intersections	Accuracy	Real Scenario implementation
[27]	Accelerometer is used to detect accident	Single point failure	Accuracy	GPS and GSM modem
[28]	Mobile phone is used to detect accident	3rd Party involvement	Response time	ION google gadget
[29]	GPS and Accelerometer are used to detect accident	Single point of failure	Accuracy	Real Scenario implementation
[16]	Alarm system with accident detection	Single point of failure	Detection time	Simulation tool
[30]	Position of vehicle is used for accident detection	Single point of failure	Accuracy	Real Scenario implementation
[31]	Detection of shortest path and accident	Single point of failure	Reliability	Simpulation tool
[32]	Reporting system with accident detection	Resources are not calculated	Accuracy	Real Scenario implementation
[33]	Vector machine is used to detect accident	Not valid as rescue system	Efficiency	Used the data of real world traffic
[34]	Detect the closed emergency point	Not respone in real time	Response time	Real Scenario implementation
[35]	Two sensors are used to detect the accident	Failure at single point	Accuracy	Real Scenario implementation
[36]	Speed factor is used to detect accident	Failure at single point	Response time	GPS and GSM modem
[37]	Air bags are used to detect accident	Contact only emergency facility	Accuracy	Real Vehicles
[27]	Reporting system with accident detection	Only one sensor is used	Accuracy	Implementation with Aurdino
[38]	Reporting system with accident detection	Reporting is not accurate	Response time	Testbed
[39]	Collision detection and infomation system	Responed to only one contact number	Response time	Real Scenario implementation

**Table 2:** ADSim Details.

Parameters	Noise	G-Force	Pressure	Speed
Threshold	140 dB	4.00 G	350 P	22–24 km/h
At Start	0	0	0	0
Scale	130–150 dB	1–100	300–400 P	1–10 G

## Data Availability

Data is available upon request.

## Sample Availability

Samples of the compounds are available from the authors.
